# *“We restrict certain things”*: a cross-sectional study of health provider adherence to WHO’s recommendation for intrapartum oral intake of fluid and food in Greater Accra, Ghana

**DOI:** 10.1186/s12884-024-06581-1

**Published:** 2024-05-30

**Authors:** Benjamin Stephens, Pearl Nanka-Bruce, Habtamu Fekadu Lashtew

**Affiliations:** 1 MOMENTUM Country and Global Leadership, Washington, DC USA; 2 MOMENTUM Country and Global Leadership Ghana, Accra, Ghana

**Keywords:** Intrapartum oral fluid and food intake for low-risk women, Positive childbirth experience

## Abstract

**Background:**

Since 2018, WHO recommends oral fluid and food intake for low-risk women during labor to enhance positive childbirth experience and respect for women’s preferences. This study investigated the current practices related to intrapartum oral intake among maternity care providers and women in public health facilities in Greater Accra, Ghana, and explored barriers and opportunities for adherence to the WHO guidance.

**Methods:**

We used a mixed-method design at five public health facilities in Greater Accra, Ghana, which included structured interviews with 11 facility-level quality improvement staff and 12 maternity care providers; a knowledge, attitudes, and practices survey with the same providers; and a client survey with 56 inpatient postpartum women. We conducted descriptive and inferential statistics, including *z*-tests to assess independent and dependent variables, and inductive thematic analyses.

**Results:**

Provider adherence to the WHO recommendation varied, with many imposing restrictions on oral intake during labor. Concerns included potential complications like Mendelson’s syndrome, consequently framing oral intake decisions as clinical and leading providers to limit women’s involvement in their care decisions. Within our sample, 54% and 43% women reported their provider counseled them on oral fluid and food intake respectively, while 41% and 34% reported their provider asked them their preference for drinking and eating respectively. Ultimately, 73% drank fluids and 19% ate food during their labor. Counseling significantly correlated with women’s intake practices (*p* < 0.01) and providers’ inquiry to women’s preferences for drinking and eating (*p* < 0.001) during labor.

**Conclusion:**

Adherence to evidence-based practices for intrapartum oral intake among low-risk women was inconsistence. Maternity care providers play a vital role in involving women in their care decisions and respecting women’s preferences. Strengthening national-level labor care guidelines and provider quality improvement approaches like in-service training, supportive supervision, and job aides to include the WHO recommendation will help providers adhere to the guidance and contribute to promoting a positive childbirth experience for women.

**Supplementary Information:**

The online version contains supplementary material available at 10.1186/s12884-024-06581-1.

## Background

Through the United Nation’s Sustainable Development Goals and Global Strategy for Women’s, Children’s, and Adolescents’ Health 2016–2030, the global health and development agenda has committed to the health and wellbeing of everyone and to reaching women, children, and adolescents through primary health care. Specific to maternal health, progress among selected indicators shows promise: between 1990 and 2015, global maternal mortality nearly halved, decreasing by almost 44% [1] and by 2022, the median national coverage among 97 low- and middle-income countries (LMICs) for births attended by a skilled provider was 96% [2]. While many maternal health challenges persist and progress remains uneven, these achievements have in part galvanized global commitments to look beyond the focus of ensuring women’s and children’s survival, to aspire to more human rights-based goals such as the delivery of women-centered care.

As part of its response to this higher calling, the World Health Organization (WHO) released its Recommendations for Intrapartum Care for a Positive Childbirth Experience in 2018 to promote “the delivery of a package of labour and childbirth interventions that is critical to ensuring that giving birth is not only safe but also a positive experience for women and their families” [3]. In this document, the WHO conditionally recommends oral fluid and food intake during labor for women at low risk (Table 1). This guidance builds upon a previous, initial recommendation that summarized the global evidence informing its decision [4]. The reviewed evidence, extracted from a Cochrane systematic review including five trials of more than 3,000 women conducted in the United Kingdom (three trials), the Netherlands (one), and Canada (one), found no benefit on key clinical outcomes for restricting oral intake among low-risk women during labor. The review also found that cases of Mendelson’s syndrome were very rate, and that while the practice of restricting food and fluid had become commonplace since the 1940s, the evidence no longer supported such practice (particularly so with the advent of more modern anesthetic techniques) [5]. As such and in support of a positive childbirth experience, WHO made its recommendation for intrapartum oral intake for low-risk women, emphasizing the respect for women’s wishes as justification [3].

**Table 1:** WHO recommendation for intrapartum oral intake for low-risk women

WHO’s positive recommendation for oral fluid and food intake during labor for low-risk women: A summary of evidence [3]
✓ Restriction of oral fluid and food intake has no beneficial effects on important clinical outcomes
✓ Emphasis is placed on respect for wishes of the woman
✓ There were no cases of Mendelson’s syndrome (inhalation of food and drink from the stomach into the lungs during general anesthesia) in more than 3,000 women participating in trials included in the systematic review

Despite global efforts to expand support of positive childbirth experiences in LMICs, there is little to no evidence from these regions pertaining to the adherence of the WHO’s recommendation on intrapartum oral intake among low-risk women. The U.S. Agency for International Development (USAID)’s MOMENTUM Country and Global Leadership (MOMENTUM) project responded to this information gap by conducting a formative assessment to review the current state of intrapartum oral intake practices in public hospitals in Greater Accra, Ghana, to contribute to the evidence for operationalizing the WHO’s recommendation.

Over the past two decades, Ghana has experienced improvements in key maternal health measures. From 2003 to 2022, institutional deliveries increased 32 percentage points, from 44 to 86%, while home deliveries declined from 56 to 13%. Between the same period, Ghana’s rate of deliveries attended by a skilled health provider increased from 55 to 87%. Despite these improvements, there remain significant variations among regions within Ghana: in 2022, while 92% of institutional deliveries occurred in Greater Accra region, only 67% occurred in the Oti region [6]. As Ghana continues to improve its institutional delivery rate across the country, there is an opportunity to reach more women with quality, women-centered maternal care services, including respect for low-risk women’s wishes on drinking and eating during labor.

## Methods

### Aim

This study had two objectives: to assess the current practices pertaining to intrapartum oral intake of food and fluid for low-risk women presenting for delivery at public healthcare facilities in Greater Accra, Ghana; and to explore how adherence to the WHO recommendation on intrapartum oral intake be supported and/or strengthened. We applied a cross-sectional, convergent parallel, mixed methods study design to answer these objectives.

### Region selection and study participant characteristics

In consultation with the Ghana Health Services (GHS), we purposively selected five public hospitals (four secondary and one tertiary) in Greater Accra, Ghana, using inclusion criteria such as facility level, geography (e.g., urban/peri-urban), monthly client volume, and post-delivery discharge practices. The participant population included facility-level quality improvement (QI) staff, maternity care providers, and inpatient postpartum women. We systematically sampled inpatient postpartum women (*n* = 56) and purposively sampled maternity care providers (*n* = 12) and QI staff (*n* = 11). Eligibility criteria for postpartum women were that they must have had a natural delivery (i.e., vaginal delivery without use of pain medication), they must have arrived at the facility during latent labor and been in the postpartum ward for at least six hours post-delivery.

### Procedure

Our study utilized both quantitative and qualitative methods to investigate our objectives, including a desk review of national, district, and facility guidelines and protocols for labor and delivery; key informant interviews with facility QI staff and maternity care providers; a knowledge, attitudes, and practices (KAP) survey with the same maternity care providers; and a client survey with inpatient postpartum women. We developed new data collection tools due to the unique and relatively unexplored focus of the study and had them content validated by maternal health and nutrition experts. A third-party research firm piloted the data collection tools, took informed consent from participants, and collected data.

### Data collection and analysis

Data collection occurred within private settings in the sampled hospitals between June – July 2022. Interviews and surveys were administered in English and Twi. Enumerators used surveys and collected responses in Kobo Collect, and audio recorded interviews, which were subsequently transcribed and translated as appropriate. Our study used a convergent parallel design, whereby the quantitative and qualitative strands occurred simultaneously and independently, and had a results point of integration. For the quantitative strand, we first conducted descriptive statistics to understand distribution, central tendency, and variability; we then conducted inferential statistics, including *z*-tests to compare proportions between independent and dependent variables. The significance threshold was set at α = 0.05. The independent variable of inquiry was whether or not women received counseling on oral intake of fluid and food during labor, and the dependent variables were whether the women either drank or ate during labor, and whether the women reported their maternity care provider asked them their preferences for drinking or eating during labor. Both sets of variables were measured using the client survey. For the qualitative strand, we conducted inductive thematic analyses, whereby we developed codes and subsequent themes and grouped them for pattern identification and aligned them with the research questions. Once both quantitative and qualitative strands had been analyzed independently, we brought them together to examine common themes, further iterate and group them, and to triangulate findings across sources, noting key points of convergence and divergence across datasets.

The study team included the MOMENTUM global nutrition and monitoring, evaluation, and learning advisors, the MOMENTUM Ghana program leadership and operations staff, and a third-party Ghanaian research firm. Ethical review and approval for this study was provided by Johns Hopkins Bloomberg School of Public Health Institutional Review Board (IRB No: 19786) and the Ghana Health Services (GHS) Ethical Review Committee (GHS-ERC 018/12/21).

## Results

### Participant demographics

We interviewed a total of 23 health care workers, including 11 facility-level QI staff and 12 maternity health providers. Among the 11 QI staff, seven were midwives, two were nurses, and there was a doctor and a QI officer. These professionals had served in their capacities between fewer than three months and 11 years, with a median length of service of 4.7 years. Among the 12 maternity care providers, nine were midwives, and there was an assistant clinical officer, a doctor, and a medical officer; all were women. They had served in their capacities between 2.5 and 12 years, with a median length of service of 6.3 years. Eight reported overseeing maternal health services at their respective facilities.

We surveyed 56 inpatient postpartum women. The mean age was 30.2 years, and there were 53 women from Greater Accra, and one each from Ashanti, Upper East, and Central regions. Women had a range of completed education levels: four had none, six had basic, 23 had junior high, 12 had senior high, and 11 completed tertiary schooling. Parity status prior to the current pregnancy was 14 nulliparous, 38 multiparous (1–4), and four grand multipara (5 or more).

### Guidelines and protocols

We reviewed more than 10 relevant policies, guidelines, and protocols at national, district, and facility levels to assess the extent to which these clinical resources incorporated the WHO recommendation for oral intake during labor for low-risk women. Our review examined influential national documents such as the Ghana National Safe Motherhood Protocol, as well as key facility-level tools like the Protocol for Admitting Clients in Labour. While some resources included related recommended practices like monitoring fluid intake as part of clinical management, none specifically incorporated the WHO recommendation or discussed fluid intake in terms of respecting the woman’s wishes as part of promoting a positive childbirth experience. It should be noted that selected resources, including the National Safe Motherhood Protocol (2008) and the National Reproductive Health Service Policy and Standards (2014), predate the 2018 WHO recommendation.

QI staff and maternity care providers confirmed the absence of the WHO recommendation within national guidance and facility tools. Of the 23 health workers interviewed, no one expressed awareness of the WHO recommendation itself or identified its presence within guidelines or job aides. As one midwife stated, “*No, I haven’t seen any guidelines concerning the intake of fluids and whatever during labor; I have not seen WHO guideline.*” Another midwife expressed a similar conclusion: “*Unfortunately, we don’t have any checklist or protocols providing us with the guidance based on food or fluid intake for pregnant women during labor.”* In a couple of instances, health workers referenced a checklist used to monitor fluid intake during labor. However, they described this more as a tool to help manage hydration rather than one to promote fluid intake as a component of positive childbirth experience. No health worker referenced any guidance or tool that included food intake.

While health workers did not have guidelines or protocols to help them apply the WHO recommendation, many expressed an interest in having them so they could provide better quality care. One maternity care provider reflected, *“We, the service providers, need to let them know that we need the guideline so that immediately, we just glance at it, even if you have forgotten, it will remind you.”* QI staff reflected on how having the guidance on the WHO recommendation would help them to their job. One senior nurse explained,

“*I am unaware of the WHO guidelines, so I would advocate that it is being brought to bed so that we would know exactly what it entails. And when we are aware of it, quality insurance* [sic] *as they say, we would make sure that we adhere to the guidelines and abide by the guidelines.”*

When asked for her recommendations, a QI staff responded,

*“It’s just the guideline. It will make the dissemination of information very easier because everyone is aware that this is what WHO and MOH, GHS have. So, implementation becomes easy. We would be able to implement it and then get the needed results.”*

Many health workers did not want to miss important maternal care guidance.

### Maternity care provider knowledge

Table 2 shows that maternity care providers had mixed knowledge about the WHO recommendation and the clinical evidence supporting it. While 8/12 (67%) providers correctly answered the question about the WHO’s recommendation for oral fluid intake, only 4/12 (33%) did so for oral food intake. One third (4/12) of providers were unsure about the WHO recommendation for either oral fluid or food intake. Such mixed results suggests a lack of familiarity with the WHO recommendation, which health worker interviews reinforced when they articulated the lack of available guidance on the topic. There was confusion about whether restricting oral intake has any beneficial effects on health outcomes. Only 7/12 (58%) providers correctly answered that restricting oral fluid intake does not have any beneficial effects on health outcomes and 5/12 (42%) correctly did so for oral food intake. One knowledge variable for which providers scored better is the recognition that restricting oral intake does not help to prevent the need for cesarean section, with 10/12 (83%) and 9/12 (75%) providers correctly responding for fluid and food, respectively. However, many did not know that ingesting fluid or food during labor does not put a woman at risk of inhalation from the stomach into the lungs during general anesthesia, with only 5/12 (42%) and 2/12 (17%) providers correctly answering for fluid and food, respectively.

**Table 2:** Knowledge survey results for maternity care providers, *n *= 12

Knowledge variable / question	Correct	Incorrect	Unsure
The WHO recommends oral intake for low-risk women during latent labor			
Fluid	8 (67%)	0 (0%)	4 (33%)
Food	4 (33%)	4 (33%)	4 (33%)
Restricting oral intake for low-risk women during latent labor has no beneficial effects on health outcomes			
Fluid	7 (58%)	5 (42%)	0 (0%)
Food	5 (42%)	7 (58%)	0 (0%)
Restricting oral intake for low-risk women during latent labor does not help to prevent the need for cesarean section			
Fluid	10 (83%)	1 (8%)	1 (8%)
Food	9 (75%)	1 (8%)	2 (17%)
Women who ingest fluid or food during labor are not at risk of inhalation from the stomach into the lungs during general anesthesia [Mendelson’s syndrome]			
Fluid	5 (42%)	6 (50%)	1 (8%)
Food	2 (17%)	8 (67%)	2 (17%)

Interviews with providers shed more perspective on their topic knowledge. The most discussed theme (mentioned 17 times among 10/12 or 83% providers) was the need to restrict oral intake (particularly for food) as a precautionary measure in the event the woman’s condition migrated from low- to high-risk and she required a cesarean section. They frequently described labor as dynamic, with an unknown outcome, and articulated restriction out of an abundance of caution. As one midwife stated, “*During the first stage of labor, we restrict certain things…like food intake…because you don’t know if the woman’s labor would not progress as expected and would end up CS* [cesarean section]*.*” Several providers further mentioned risk of aspiration in the event a woman had previously consumed food during labor, but soon thereafter required surgery. For example, one midwife stated, *“So based on how you give them* [women] *information, they would realize that, if you take the food and it happens you are being sent for cesarean section, it can cause choking and no woman would want to be choked with food*.” Fear of the risk of Mendelson’s syndrome was real for some providers, as one midwife explained,

*“Because in labour, you can’t tell the outcome, so we do counsel them as to what to eat during that time, during their labor because when you eat heavily…and then your labour doesn’t go well, and you end up going for CS* [cesarean section] *the anesthetist wouldn’t like to give the anesthesia because he cannot do it whiles you have taken that heavy food.”*

This concern provides context to how the majority of providers (8/12 or 67%) answered incorrectly or were unsure (2/12 or 17%) about the knowledge survey question on the same topic (see Table 2). These concerns frequently influenced the type and amount of fluid and food providers permit low-risk women under their care to consume during labor.

Nonetheless, some providers also expressed the clinical importance for encouraging drinking and eating – albeit in measured amounts and types – with six (50%) and seven (58%) of 12 providers discussing hydration and energy for delivery needs respectively. One midwife with this viewpoint stated, “*Fluid intake during the first stage of labor, as I said, is important because when the woman is dehydrated, it would end up with her being weak during the second stage.*” Providers generally understood the clinical benefits of drinking and eating during latent labor as means to prepare the woman for the active stage.

### Counseling on oral intake of fluids and foods

**Fig. 1:**
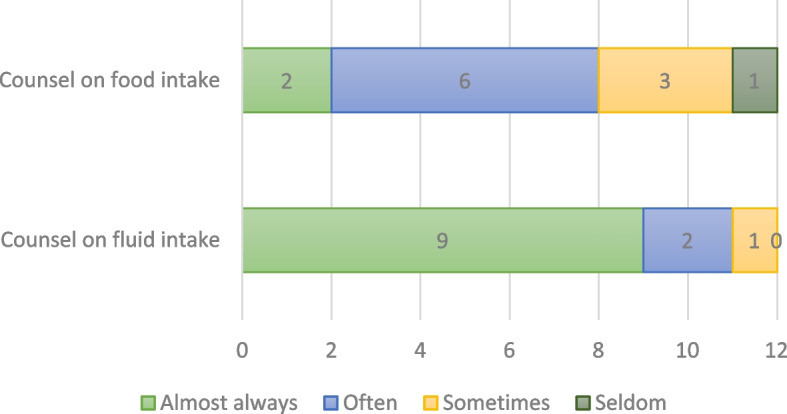
Provider-reported practices: Counsel low-risk women on oral intake during labor, *n*=12

Study findings show mixed results on whether providers counseled low-risk women on oral fluid and food intake during labor, with providers and postpartum women reporting inconsistent experiences. Providers reported frequently counseling women on the importance of oral fluid intake in particular, and to a lesser extent, food intake. As Fig. 1 (Provider-reported practices: Counsel low-risk women on oral intake during labor, *n* = 12) shows most providers reported they almost always or often counsel on drinking fluids (11/12, 92%), and many reported they almost always or often counsel on eating foods (8/12, 67%). Few reported they sometimes or seldom counsel for drinking fluids (1/12, 8%) and eating foods (4/12, 33%). Interview data supported these results, with most providers discussing how they encourage drinking and eating. *“We try as much as possible to educate them* [women] *on the needs, importance, and the reasons why they should take in food and fluids during labour,”* asserted one midwife, while another specified, *“the pregnant woman need* [sic] *a lot of energy to push during labour, so encourage women to take in more fluids and then take in food in order for her to get the energy to push during labour.”* There was no clear link between providers’ length of professional service and counseling practices. Those who reported to almost always (9/12) or often (2/12) counsel women on drinking had an average length of service of 5.7 and 4 years, respectively, while those who reported to almost always (2/12) or often (6/12) counsel women on eating had an average length of service of 3.5 and 8.6 years, respectively (data not shown).

**Fig. 2:**
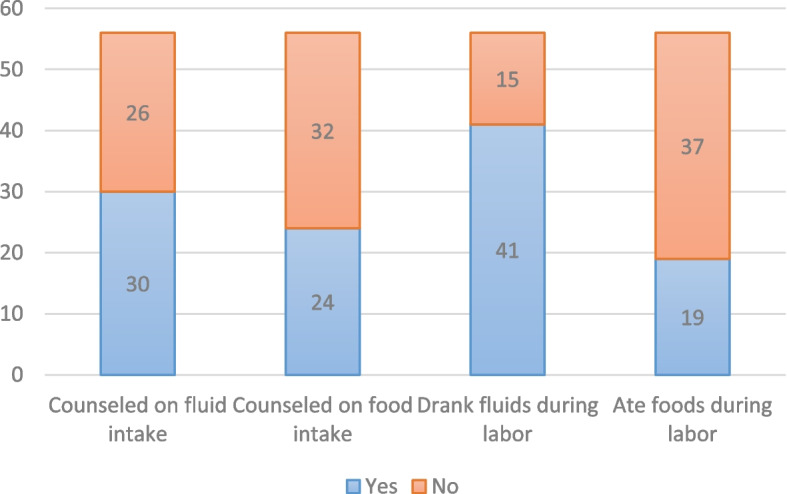
Women-reported experiences: Oral intake counseling and practice during labor, *n*=56

However, postpartum women reported more varied counseling experiences with providers. Figure 2 (Women-reported experiences: Oral intake counseling and practice during labor, *n* = 56) shows whether postpartum women received counseling on oral intake and if they decided to drink or eat during their labor. Of the 56 surveyed women, approximately half (30, 54%) reported their provider counseled them on fluid intake, while fewer (24, 43%) reported so for counseling on food intake. Ultimately, 41 women (73%) drank fluids during their labor, while only 19 (34%) ate food.

**Fig. 3:**
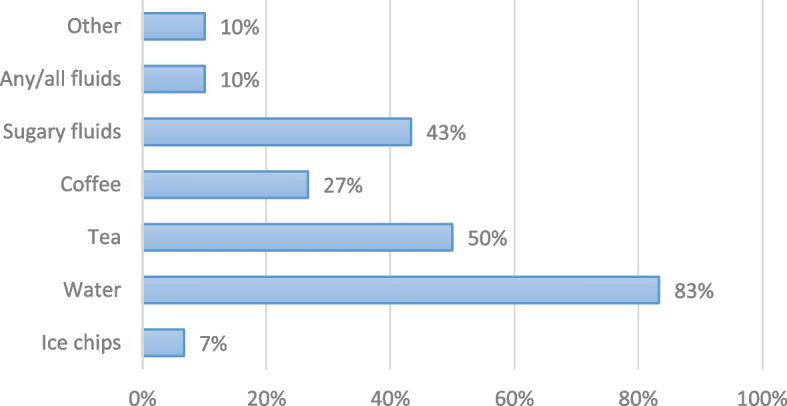
Women-reported experiences: Types of permissible fluids, as counseled by provider, *n*=30

An examination of the counseling content adds depth to our understanding of provider practices. The women who received counseling on fluid intake (*n* = 30) reported which types of fluids their provider said were permissible. Figure 3 (Women-reported experiences: Types of permissible fluids, as counseled by provider, *n* = 30) shows that 25/30 (83%) counseled women reported their provider said water was permissible, yet only 3/30 (10%) said their provider counseled that any/all fluids were permissible (guidance which is consistent with the WHO recommendation). This bias towards water and other clear fluids also emerged as a sub-theme in interviews with providers. As one midwife stated, “*For the low-risk women…we encourage them to take in much fluid, so far as it is clear*.” Another midwife claimed, “*When they* [laboring women] *come, we encourage them to take more fluid like water to prevent dehydration*.” Provider attitude data reinforces this, with all 12 maternity care providers reporting they either strongly (9/12, 75%) or somewhat agree (3/12, 25%) that intake of water is safer than other types of fluids during labor (data not shown).

**Fig. 4:**
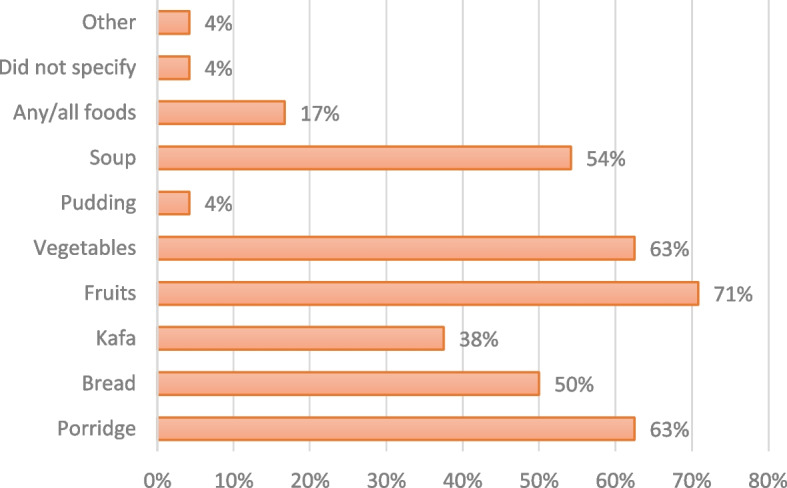
Women-reported experiences: Types of permissible foods, as counseled by provider, *n*=24

There was variance among the types of foods maternity care providers told women were permissible during counseling, as shown in Fig. 4 (Women-reported experiences: Types of permissible foods, as counseled by provider, *n* = 24). Among the women counseled on food intake (*n* = 24), most reported their provider counseled them on lighter foods like fruits (17/24, 71%), vegetables, and porridge (15/24, 63% each). Yet only 4/24 (17%) women reported being told by their provider that any/all foods were permissible (guidance that is consistent with the WHO recommendation). Provider interviews further reinforced postpartum women experiences on being told which types of foods were permissible. Several providers described some of the reasoning for how they counsel low-risk women on food intake. One midwife stated, “*We do light* [food] *even though they are low risk. We do light meal because you don’t know how the labor might progress…so we try and do light meal and we avoid the heavy meals*.” Another midwife relayed a similar logic: “*I prefer them* [laboring women] *taking in something light…but if they really eat solid foods, that can take hours before it digests, and then there is an emergency, it means they would have to intervene before they take you in, if they want to prevent aspirations.”*

Counseling emerged as an effective practice to encourage oral intake among laboring women. Data from the client survey showed a strong correlation between counseling status and the practice of drinking or eating during one’s labor. We conducted Z-tests for two proportions to examine the relationship between counseling status and the practice of drinking or eating. Table 3 summarizes these results among 56 low-risk women. Those who were counseled by their maternity care provider on fluid or food intake were statistically more likely to drink (*P* = 0.0026) or eat (*P* = 0.0067), than not.

**Table 3:** Counseling status correlation with oral intake practice and provider inquiry of women’s preference for oral intake, *n *= 56

Indicator / Measure	Freq	Prop	Diff	Significance Level
Woman counseled on fluid intake and drank	27/30	90%	36%	*P* = 0.0026^a^
Woman not counseled on fluid intake and drank	14/26	54%		
Woman counseled on food intake and ate	13/24	54%	35%	*P* = 0.0067^b^
Woman not counseled on food intake and ate	6/32	19%		
Woman counseled on fluid intake and asked her preference for drinking	23/30	77%	77%	*P* = 0.0001^c^
Woman not counseled on fluid intake but asked her preference for drinking	0/26	0%		
Woman counseled on food intake and asked her preference for eating	15/24	63%	44%	*P* = 0.0009^d^
Woman not counseled on food intake but asked her preference for eating	6/32	19%		

^a^95% CI: 10.23%–55.02%; Chi-squared: 7.622; DF: 1

^b^95% CI: 9.72%–55.54%; Chi-squared: 7.353; DF: 1

^c^95% CI: 55.23%–88.44%; Chi-squared: 33.468; DF:1

^d^95% CI: 18.16%–63.03%; Chi-squared: 11.084; DF:1

### Provider respect for women’s preference on oral intake

**Fig. 5:**
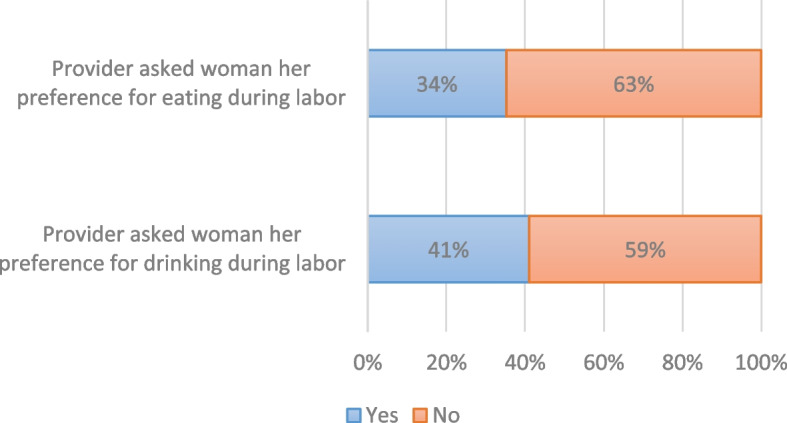
Women-reported experiences: Provider asked her preference for oral intake, *n*=56

Postpartum women reported their experiences about providers asking them their preference for oral intake during labor, shown in Fig. 5 (Women-reported experiences: Provider asked her preference for oral intake, *n* = 56). Many disclosed that their provider did not ask their preference for either drinking (33/56, 59%) or eating (35/56, 63%). We examined the relationship between counseling status and whether the provider asked the woman her preference for drinking and eating. Results from Z-tests for two proportions showed a strong correlation between the two variables (see Table 3). According to the 56 women surveyed, providers who counseled them on fluid or food intake were statistically more likely to ask the women their preferences for drinking (*P* = 0.0001) and eating (*P* = 0.0009), then not. When providers reflected on whether and to what extent they respect the woman’s preference or oral fluid or food intake, there were conflicting accounts. One midwife offered her support, *“during the first stage of labour, since they have not gotten into active phase, they can take in any food of preference, any food they prefer and any beverage they feel like taking.”* Yet others did not share this perspective: One midwife explained, as she reflected on some women's desire to eat during labor,

*“some* [women] *will come, you would explain to them, they wouldn’t understand. They want to do what they seem right to them, but we need to explain to them, for them to understand that, maybe at that particular moment she doesn’t need whatever she thinks is good for her, so we need to counsel for them to understand.”*

Another midwife reinforced this point about needing to override women’s preferences:

*“They* [women] *have the preferences to eat because some people naturally during pregnancy eat a lot. So when you restrict them a little while, they think you are starving them and starving the baby too…when you educate them on fluids, eating, they think you are starving them and starving the baby, even though sometimes we tend to get them to understand, not all of them appreciates that.”*

**Fig. 6:**
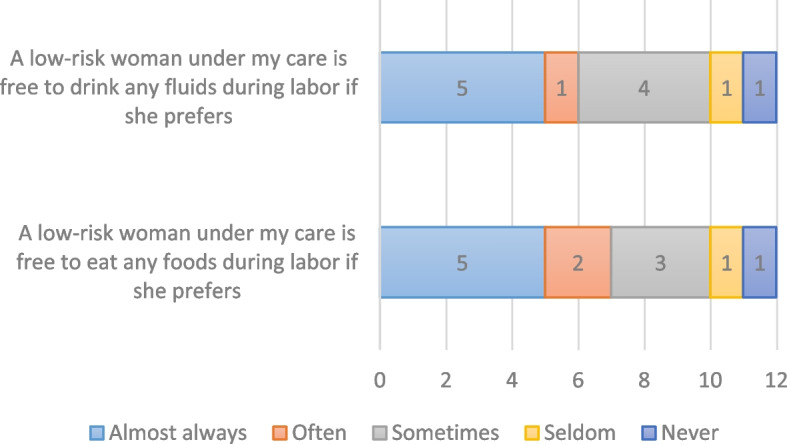
Provider-reported practices: Low-risk women freely decide what to drink or eat during labor, *n*=12

Maternity care providers described their perspectives on decision-making about oral intake for low-risk women, and to what extent women under their care are free to drink or eat during labor. Figure 6 (Provider-reported practices: Low-risk women freely decide what to drink or eat during labor, *n* = 12) shows how providers self-reported their practice of permitting a low-risk woman to freely drink or eat during labor, with half (6/12, 50%) reporting they almost always or often let women freely drink any fluids if they prefer and slightly more than half (7/12, 58%) reporting the same for letting women freely eat any foods if they prefer. During interviews, however, providers more consistently articulated their view that medical professionals – and not women – have final decision-making authority on oral intake during labor, with 8/12 (67%) providers claiming the provider decides exclusively (data not shown). When asked about who makes this decision, one midwife described, *“When the case is a normal case and then is a low risk, when the person comes, you, the midwife, can decide.”* Another midwife had a similar perspective, claiming, *“Midwives can also make the final decision, or let me say, the doctors or the specialist. Or let me say, the person taking care of that laboring woman makes the final decision.”* On one rare occasion, a midwife offered a different perspective: “*If the woman tells me she wants malt, I would not say no, take in water instead if it’s low risk, because I know there is no risk. If she wants food, why not, she would eat.”* Yet for most providers, they viewed medical professionals as the final decision-making authority on oral intake for low-risk women during labor.

## Discussion

To our knowledge, this is the first study of its kind that investigated adherence to the WHO recommendation on intrapartum oral intake for low-risk women in a LMIC setting. Our research adds to the growing body of evidence on positive childbirth experience and contributes unique insight to contextual challenges and enablers for adherence to the WHO recommendation. We applied a mixed methods approach to understand the current state of intrapartum oral intake practices in public hospitals in Greater Accra, Ghana. Our findings show that maternity care providers at study sites are, practically speaking, not implementing the WHO recommendation for intrapartum oral fluid and food intake for low-risk women.

With the evolution of the global health agenda to prioritize women-centered care, positive childbirth experience has become a maternal and newborn health goal unto itself. This broader, more encompassing approach to care weighs the emotional and psychological wellbeing of the woman as much as the physiological process as essential components of high-quality labor and childbirth care. Such is reflected in the WHO recommendations for intrapartum care, whose guiding principles affirm that labor and childbirth should be individualized, woman-centered, and with the objective of supporting a positive childbirth experience [7].

WHO places women’s wishes as central to its recommendation for intrapartum oral intake [3]. Research shows that women value having a sense of control and being involved in decision-making about their care during labor, and maternity services should be responsive to those needs [8, 9]. When women are involved in decision-making about their own care, they tend to express feelings of trust, safety, comfort, and control during their labor, thereby contributing to a more positive experience [10–12]. Our study showed maternity care providers limited women’s involvement in their care decisions with respects to intrapartum oral intake by rarely asking them about their preferences and inconsistently and incompletely counseling them on their options for oral intake. Many providers viewed decisions about intrapartum oral intake as part of their clinical authority and relied on out-of-date clinical knowledge about risk factors when making decisions about whether and what a low-risk woman could drink or eat. Respect for the women’s wishes seemingly did not factor high in how maternity care providers approached intrapartum oral fluid and food intake.

This study reveals certain challenges to the promotion of a positive childbirth experience by identifying barriers that prevent low-risk women from freely drinking or eating during their labor. Maternity care providers lacked clear guidance to help them navigate the WHO recommendation or to consider oral intake as a facet of women-centered care. This is further compounded by providers’ perceived fear of the complications, including the risk of Mendelson’s syndrome – despite evidence showing this condition to be exceedingly rare [5]. They often did not prioritize asking women their preferences for oral intake and some excluded women from the decision-making process on whether and what to drink or eat during labor. These practices suggest a viewpoint that oral fluid and food intake for low-risk women is ultimately a clinical decision and not one to be made by the woman. When providers counseled women on oral intake, they often omitted choices or placed limitations on what women can and cannot consume. Many providers tended to push fluids like water and lighter foods like fruits or soup and dissuaded women from consuming heavier foods – a decision likely influenced more by an apprehension about managing a woman’s risk level throughout labor than any current clinical rationale. This may be motivated in part by providers wanting to avoid dehydration during labor, which can affect all women – even those who are low risk – and decrease the strength and frequency of uterine contractions and/or cause abnormalities in the baby’s heart rate pattern. While this study did not specifically examine how providers monitor and manage women’s risk levels during labor, the findings shed interesting perspective on how this component of care affects others like counseling. Taken together, the findings tell a story of small, yet important missed opportunities to support women’s involvement, comfort, and their sense of control during childbirth.

Nonetheless, this study also identified enablers that could be leveraged to promote adherence to the WHO guidelines if properly strengthened and supported. Counseling emerged as a powerful tool to promote individual choice and an opportunity to invite low-risk women to participate in decision-making about their childbirth experience. It also supported providers to better consider women’s preferences for oral intake during labor. Many providers recognized the clinical value of women drinking and eating during labor through their mention of the importance for hydration and having energy for the active stage of labor. We also found there is interest among providers to learn and follow the WHO guidance. During interviews, they frequently requested to have clear guidance and training on the WHO recommendation, showing enthusiasm in incorporating it within their daily routine. A strategy that harnesses and builds upon these enablers to introduce, roll out, and monitor adherence to the WHO guidelines – while strengthening other facets of care like monitoring risk for laboring women – seems well-positioned for success.

### Limitations

Our findings are limited to five secondary and tertiary facilities in Greater Accra, Ghana, where the data were collected. Much of Ghana’s regional demographic diversity and its health system’s primary care is not represented in our findings. While sampling for maternity care provider and postpartum women occurred within the same wards, it was not possible to verify if the providers who cared for surveyed women were the same ones interviewed in our study. A direct comparison between maternity care provider and postpartum women data is therefore not possible. Provider interviews took place during daytime hours only; our results do not include providers who work nightshifts or weekends. Although nutritionists or nutrition program managers are key stakeholders to champion inclusion of the WHO guidance on intrapartum fluid and food intake in the national nutrition plan and nutrition training curriculum, our study did not assess the perspectives of these health cadres, which limits our recommendation among nutrition expertise.

## Conclusion

Women’s involvement in decision-making and respect for their wishes during labor are crucial aspects of respectful maternal care and a positive childbirth experience. Maternity care providers play a vital role in facilitating this involvement and promoting this respect, and counseling serves a critical medium to do so. While this study focuses on provider practices in Greater Accra, Ghana, challenges hindering adherence to the WHO recommendation on intrapartum oral intake for low-risk women are likely widespread. Further research investigating adherence in diverse settings will build the evidence base and inform comprehensive guidance for maternity care providers. Strengthening national-level labor care guidelines and provider quality improvement approaches like in-service training and supportive supervision to include the WHO recommendation will help providers adhere to the guidance and contribute to promoting a positive childbirth experience for women.

### Supplementary Information

Additional file 1. Knowledge, Attitudes, and Practices (KAP) of maternity providers. Description: Dataset from KAP survey administered to enrolled maternity providers.

Additional file 2. Client Survey from Postpartum Women. Description: Dataset from client survey administered to enrolled postpartum women.

## Data Availability

The datasets generated by the survey research and supporting the conclusions of this article are included within the article (and its additional files).

## Abbreviations

GHS: Ghana Health Services
KAP: Knowledge, attitudes, and practices
LMIC: Low or middle-income country
QI: Quality improvement
USAID: United States Agency for International Development
WHO: World Health Organization
